# Bringing cell biology into classroom: tips to culture and observe skeletal muscle cells in high school and college

**DOI:** 10.1007/s11626-024-00906-2

**Published:** 2024-05-14

**Authors:** Ryoichi Matsuda, Fumiko Okiharu

**Affiliations:** Department of Science Education, Tokyo University of Science, 1-3 Kagurazaka, Shinjuku-Ku, Tokyo, 162-8601 Japan

**Keywords:** Skeletal muscle cell culture, Cell differentiation, CO_2_ incubator, Smartphone microscope, Cell biology education

## Abstract

Watching living cells through a microscope is much more exciting than seeing pictures of cells in high school and college textbooks. However, bringing cell cultures into the classroom is challenging for biology teachers since culturing cells requires sophisticated and expensive instruments such as a CO_2_ incubator and an inverted phase-contrast microscope. Here, we describe easy and affordable methods to culture and observe skeletal muscle cells using the L-15 culture medium, tissue culture flask, standard dry incubator, standard upright microscope, and modified Smartphone microscope. Watching natural living cells in a “Do-It-Yourself (DIY)” way may inspire more students’ interest in cell biology.

## Introduction

Skeletal muscle cells are one of the best research subjects on cell growth and differentiation. Cell biologists have been fascinated by its dynamic changes in morphology and biochemistry during muscle cell differentiation. Okazaki and Holtzer (1965, 1966) pioneered the immunofluorescent antibody technique to study muscle myosin expression at the early phase of skeletal muscle cell differentiation. Later, one of Holtzer’s students, Weintraub, and his colleagues (1987) discovered the muscle-specific transcription factor, Myo D, and elucidated the molecular cascade of muscle cell differentiation (Davis *et al*. 1987). Skeletal muscle cells may also be appropriate for studying cell differentiation in high school and college since their morphological and biochemical changes are evident, and it takes a few days to see the main part of muscle cell differentiation in culture. However, bringing cell cultures into the classroom is challenging for biology teachers since culturing cells requires specialized and expensive instruments such as a CO_2_ incubator and an inverted phase-contrast microscope. Due to these technical reasons, biology teachers avoid cell cultures in high school and college. Therefore, students learn about cells mostly from textbooks and Internet sources without any experience of watching actual living cells. Under these conditions, “Study nature, not books (Louis Agassiz)” has never been fulfilled in Cell Biology educational sites.

To overcome these difficulties, developing accessible and affordable methods to culture and observe natural living cells in the current school environment would be preferable.

The main points of the present study are as follows:

1. To avoid using a CO_2_ incubator, we switched the culture medium from Eagle’s minimum essential medium (MEM) to Leibovitz’s L-15 medium, which does not need the CO_2_ gas.
2. By culturing cells in culture flasks, not in culture dishes, we could see cultured muscle cells under a standard upright microscope by flipping over the flask upside-down.
3. Alternatively, we could observe cells using a lens taken from the laser pickup head of a used/broken compact disk (CD) player, attaching the CD lens to a “Simple Camera”–installed Smartphone—and illuminating the cells with a light-emitting diode (LED) lamp with a pinhole disk, we could focus on cultured cells at the single-cell level. We named this method “the inverted Smartphone microscopy.” Therefore, we can culture and watch natural living cells in high schools and colleges. Using these methods mentioned here, it became possible for students to culture, watch, and take pictures/movies of living cells without using expensive instruments. Watching actual living cells may inspire many students’ interests in cell biology.

## Materials and methods

### *Muscle cell culture*

We used plastic culture flasks (25 cm^2^/Tissue Culture Flask with double seal cap, code no. 3100–025, IWAKI, Japan) to replace culture dishes for muscle cell culture. The bottom surface of the flasks was coated with autoclaved 1% porcine skin gelatin solution (G1890, Sigma-Aldrich, St. Louis, MO) to enhance cell adhesion to the surface (Matsuda *et al*. 1987, Sato *et al*. 2002). Instead of using Eagle’s MEM, we used Leibovitz’s L-15 culture medium (cat no. 128–06075, Fujifilm, Japan, Leibovitz 1963). The L-15 medium supplemented with 10% horse serum (H1270, Sigma-Aldrich), 4% chicken embryo extract (cat no. 2850145, M.P. Biomedicals, Solon, OH) according to Shimada *et al*. (1967) and Yaffe (1968), and long-lasting vitamin C, l-ascorbic acid 2-phosphate at a final concentration to 0.2 mM (cat no. 66170–10-3, Fujifilm, Japan), according to Takamizawa *et al*. (2004), and a mixture of antibiotics (cat no. 168–23191, Fujifilm, Tokyo) at 0.5%. We used 0.5% antibiotics mixture instead of 1% to reduce the side effects of streptomycin (Moss *et al*. 1985). The L-15 medium contained phosphates, free amino acids, and sodium bicarbonate to keep the pH neutral without CO_2_. Using the L-15 culture medium in a culture flask, we could culture cells in the standard dry incubator for over 2 weeks at 37 °C without any medium change.

We dissected a pair of breast muscles, *M. Pectoralis major*, from 11-day chick embryos. We washed the muscle tissue in a Petri dish in calcium- and magnesium-free phosphate-buffered saline (PBS, cat no.166–23555, Fujifilm, Japan). We transferred the muscle tissue to a dry Petri dish, scissored it to make small pieces, and poured them into a 15-mL culture tube filled with PBS. We placed the tube in a rack and waited for several minutes until the tissue pieces sank to the bottom. Then, we discarded the supernatant and added 4 mL of culture medium. Muscle cells were released by repeated pipetting through a 270-mm-long Pasteur pipette mechanically, according to Hagiwara *et al*. (1985). We took special care to avoid air bubble formation. In this way, cell dissociation procedures became more manageable and quicker than classic trypsinization. Since we skipped the filtration of cell suspension, muscle tissue clamps remained occasionally in the cell suspension. However, contamination of tissue clamps, or explants, gave another opportunity to watch cells migrate outward from the tissue clamp. We could start cell culture by adding two to four drops of the cell suspension into one culture flask with 12 mL of culture medium. A pair of breast muscles was enough to prepare more than 20 culture flasks.

### Settings of inverted and standard upright microscopes for observation

**Figure 1.**
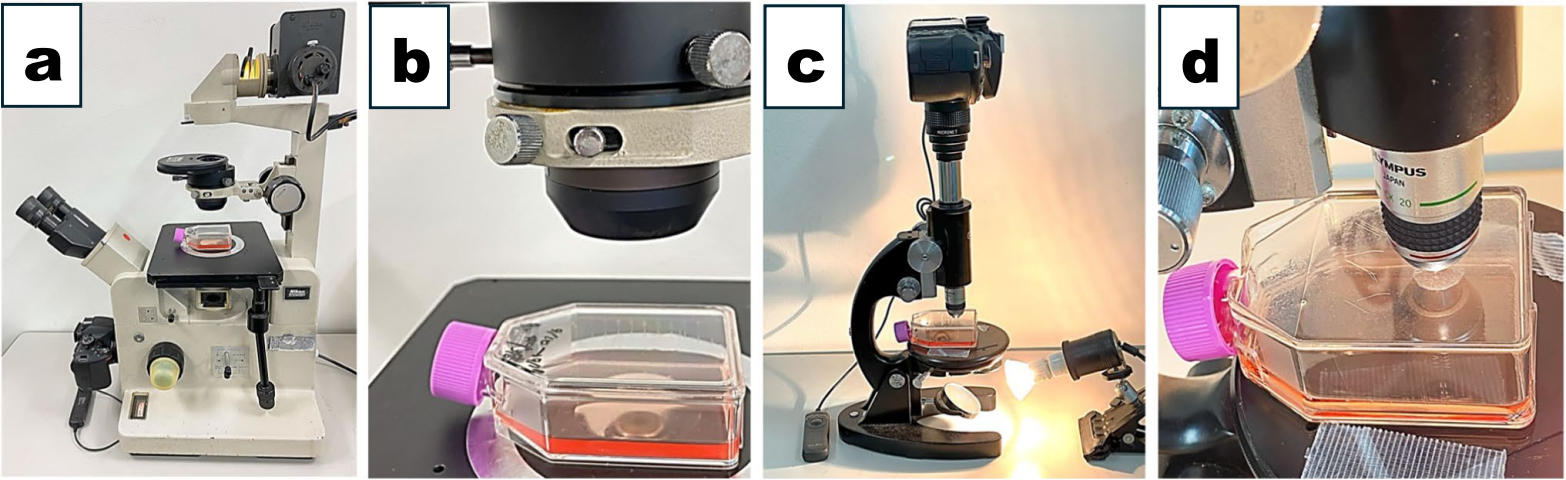
Setting up of inverted phase-contrast and non-phase-contrast upright microscopes for observation. (*a*) A culture flask placed on the stage of an inverted phase-contrast microscope. (*b*) Enlargement of the flask on the stage. Note the flask’s spout turned upward. (*c*) A flask flipped upside down and placed on the stage of a standard non-phase-contrast upright microscope. (*d*) Enlargement of the flask on the stage. Note the flask’s spout turned downward. The condenser lens aperture set the minimum.

We used an inverted phase-contrast microscope, Nikon TMD, as a control. The flask's spout turned upwards on the inverted microscope stage, as shown in Fig. 1*a* and *b*. To watch the cells under the standard upright microscope (Olympus G.K., Tokyo, Japan), we flipped the flask upside down and placed it on the microscope stage to keep the distance between cells and the objective lens at a minimum (Fig. 1*c*, *d*). In this case, the spout of a flask on the stage turned downwards. We adjusted adequate lighting by changing the aperture of the condenser lens, the angle of the reflection mirror of the microscope, and the lamp’s voltage. We used a Nikon D5100 digital camera with an ISO 1600 setting to take photographs.

### Makings of “the inverted Smartphone microscope” with a CD lens

**Figure 2.**
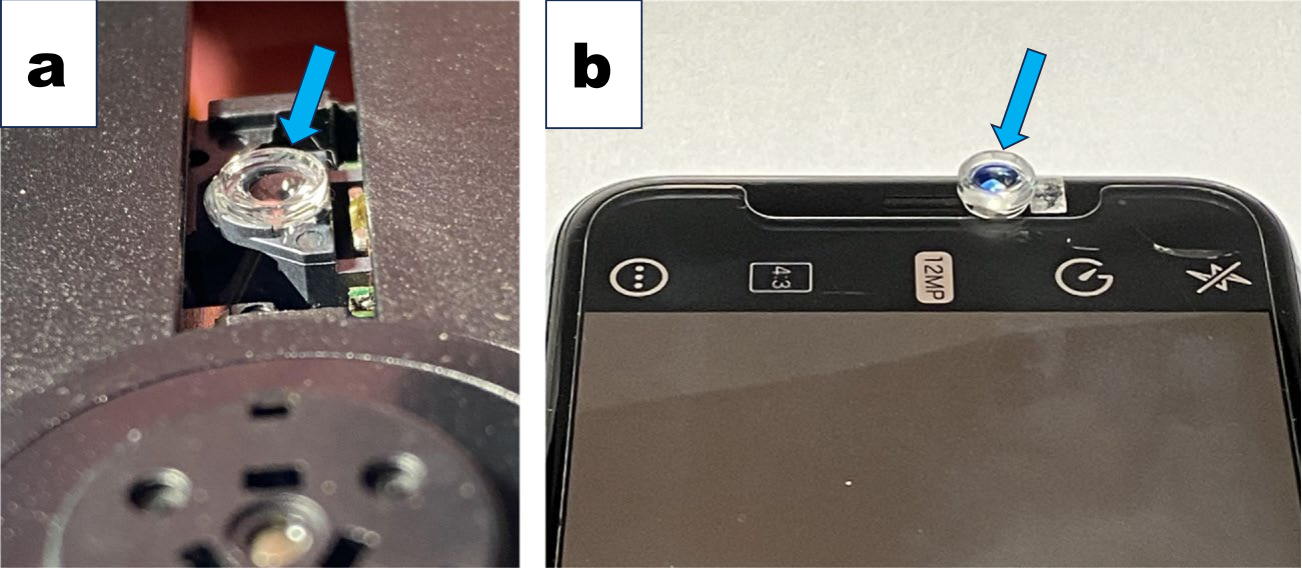
Makings of an “inverted smartphone microscope.” (*a*) The Lazer pickup head appears in a CD player. A *blue arrow* indicates the CD lens. (*b*) We attached the CD lens to the top of the selfie lens of the Smartphone, Apple iPhone 11Pro. The CD lens frame was attached close to the smartphone selfie lens with a small piece of double-sided tape. Simple Camera software was pre-installed on the Smartphone before its use.

We used a Smartphone as an alternative microscope since the penetration rate of Smartphones is much higher than that of standard microscopes in high school and college environments. According to Yoshino (2022), we utilized a single lens of a laser pickup head of a used/broken CD player to increase the magnification (Fig. 2*a*). Laser pickup heads are also available from online stores. We attached the CD single lens at the center of the Smartphone selfie lens with a small piece of Scotch double-sided tape (blue arrow in Fig. 2*b*). We installed “Simple Camera” software on a Smartphone to add an electronic zoom function to the selfie camera. We could observe cultured cells at the single-cell-level cell level with Simple Camera magnification at 10 × .

**Figure 3.**
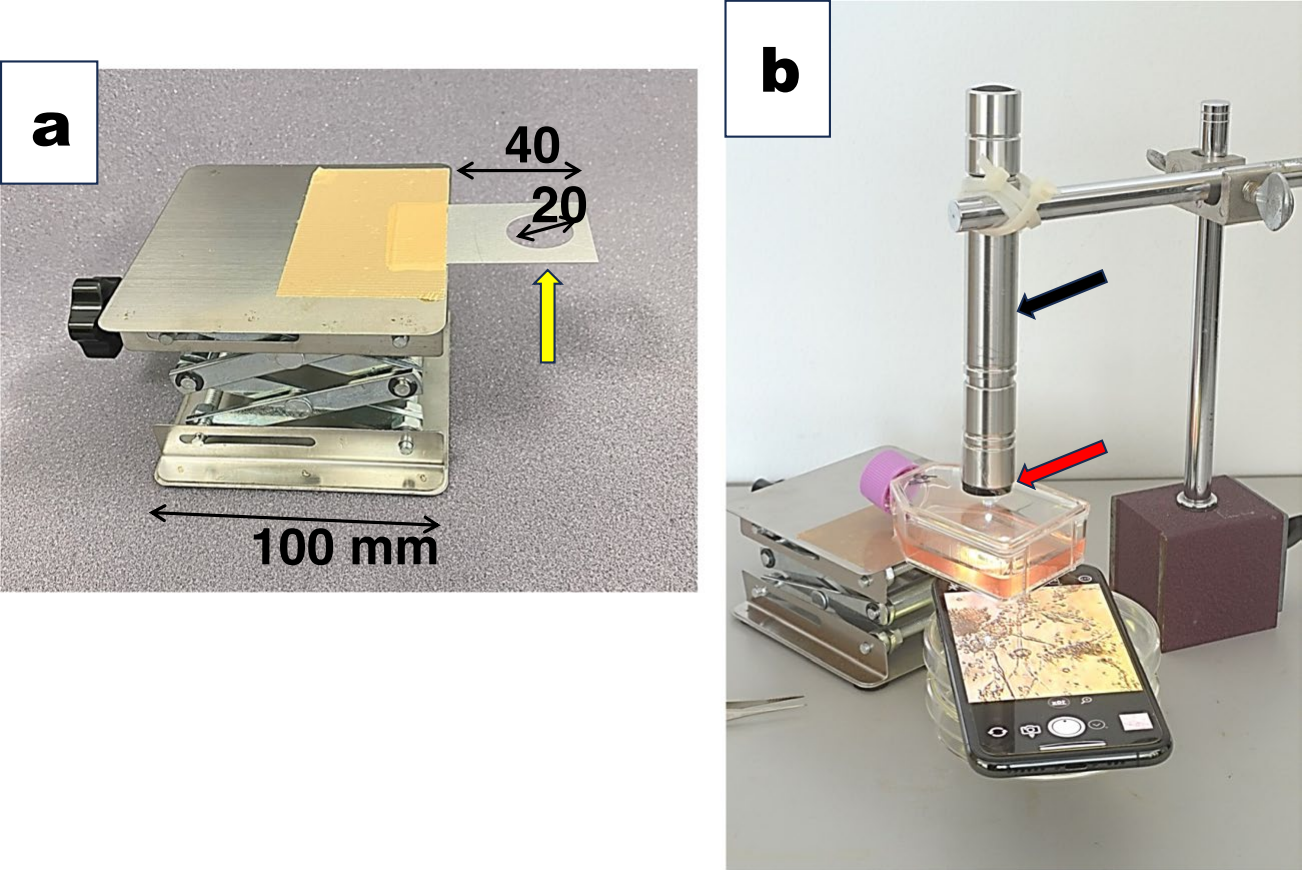
Setting the focus of the inverted Smartphone microscope using a laboratory jack. (*a*) We made an aluminum plate with a hole of 20 mm in diameter (*yellow arrow*) at the edge and attached it to a laboratory jack with duct tape. We used the hole as an observation window of the cell. The size was indicated in mm. (*b*) We placed a culture flask on the aluminum plate and inserted the Smartphone underneath the hole. An LED lamp (indicated with a *black arrow*) with a black plastic disk of a 1.5-mm pinhole was attached to the lamp aperture (marked with a *red arrow*), illuminating the CD lens’ area from above. The lab jack brought the flask into focus.

We prepared a Smartphone microscope stage with an aluminum plate, 30 mm × 100 mm × 0.5 mm in thickness, with one hole of 20 mm in diameter at the edge of the plate shown in Fig. 3. We used the hole as an observation window. We attached the aluminum plate to a laboratory jack with duct tape (Fig. 3*a*). The light-emitting diode (LED) lamp (black arrow in Fig. 3*b*) illuminated the cells. We adjusted the distance between the culture flask and the CD lens with the laboratory jack to bring cells into focus.

**Figure 4.**
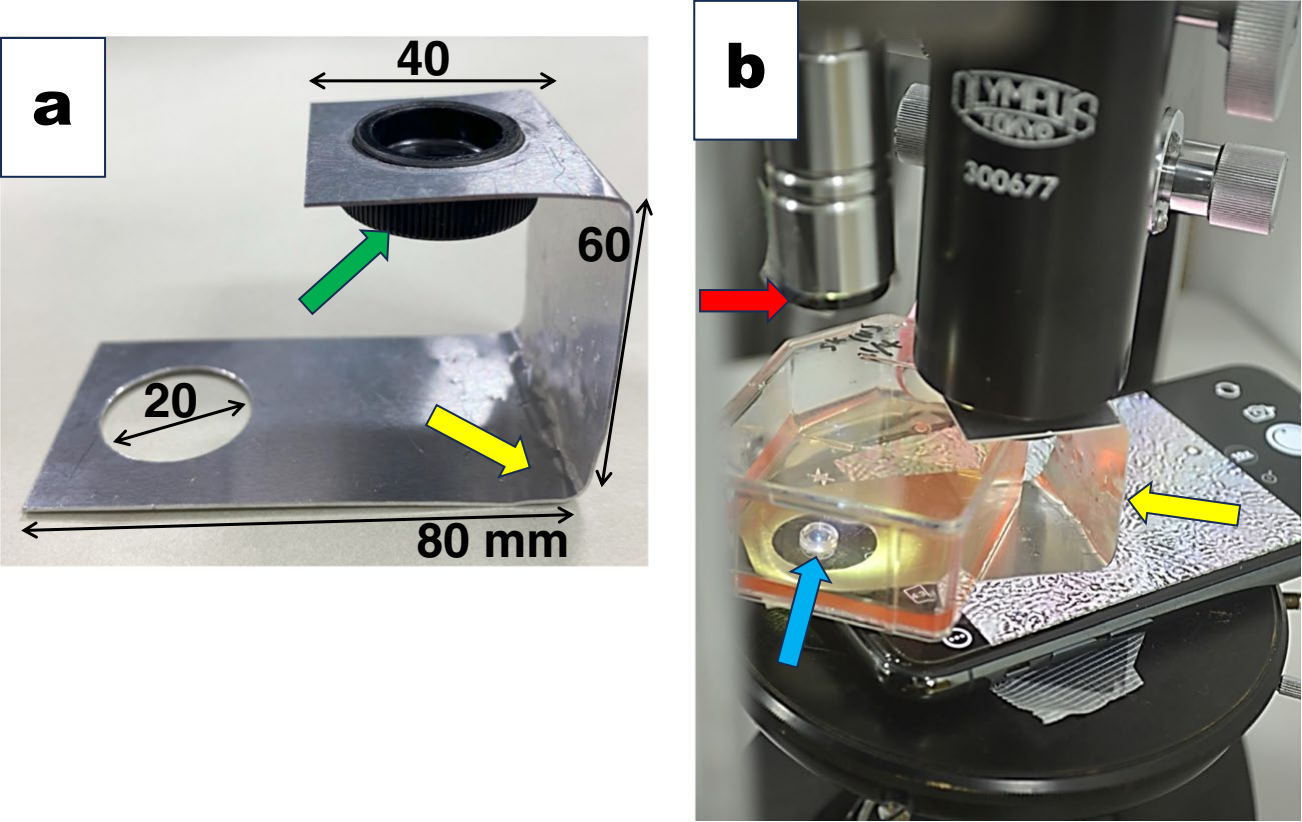
An alternative way of setting the “inverted Smartphone microscope’ and the standard microscopic barrel. (*a*) An aluminum plate, 30 mm × 200 mm × 0.5 mm thick with two 20-mm holes in diameter at both edges, was bent to form a horizontal U-shape (*yellow arrow*). We removed the objective lens from the standard upright microscope before use. We attached the upper hole to the microscope barrel with a tarry cap (*green arrow*). We used the lower hole as the observation window. (*b*) The culture flask was placed on a horizontal U-shape aluminum plate’s lower plate. We inserted the Smartphone with a CD lens underneath the lower hole of the U-shaped plate. A black plastic disk with a 1.5-mm pinhole in diameter was attached to the aperture of the LED lamp (*red arrow*). The LED light illuminated the area of the CD lens (*blue arrow*) from above the culture flask. The focusing devise of the upright microscope brought the cells into the focus of the Smartphone selfie camera.

Alternatively, we used the barrel of a standard microscope to get the cells in focus instead of a laboratory lack. We prepared a horizontal U-shaped aluminum plate, 30 mm × 180 mm × 0.5 mm, with two holes of 20 mm in diameter at both ends of the plate (yellow arrows in Fig. 4*a*, *b*). We used the hole in the lower plate as an observation window and the hole in the upper plate to attach the microscope barrel with a tarry cap (green arrow in Fig. 4*a*). We placed the culture flask on the horizontal bottom plate. And we adjusted the distance between the cells and the CD lens (blue arrow in Fig. 4*b*) with the focusing device of the upright microscope to bring cells into focus.

### Illumination of cells with LED lamp through a pinhole

We illuminated the cells with an LED lamp of 2000 lumens with or without a pinhole disk (red arrows in Figs. 3*b* and 4*b*). The diameter of the pinhole was 1.5 mm. We also used an inverted phase-contrast microscope with a Nikon D5100 digital camera to compare the image quality.

## Results and discussions

### Culture flask as a replacement for culture dish

The L-15 medium could avoid using a CO_2_ incubator and keep the medium pH neutral under a standard atmosphere (Leibovitz 1963). Repeated pipetting muscle tissue pieces to release single cells mechanically was easier than classic trypsinization. We could watch the significant processes of myogenesis, including striated myofibril formation and spontaneous contractions within 7 d in culture. The cells’ adherence to the flask’s bottom surface was strong enough to endure the vibrations during transportation. Therefore, students could take the flask and continue watching cells at home.

### Pinhole illumination improved sharpened images

**Figure 5.**
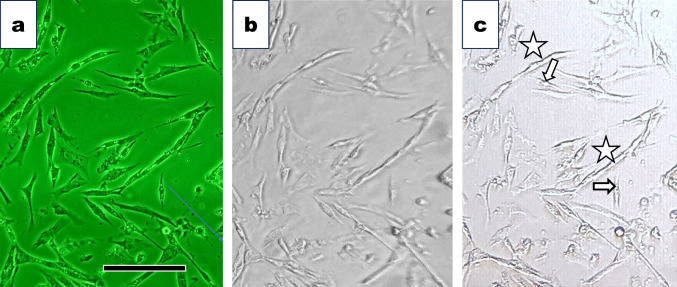
Effect of pinhole illumination on the microscopic images. (*a*) One-day cultured chicken breast muscle cells observed under the inverted phase-contrast microscope, Nikon TMD. We took the photograph with a Nikon D5100 digital camera at ISO1600 setting. The *bar* indicates 100 µm. (*b*) The same area shown in Fig. 6*a* was observed with an inverted Smartphone microscope illuminated by an LED lamp without the pinhole disk. “Simple Camera” software-installed Smartphone, Apple 11Pro, was used. We took the photograph with the smartphone selfie camera. (*c*) We observed cells in the same area shown in Fig. 6*a* and *b* under an inverted Smartphone microscope illuminated with the LED lamp through a pinhole disk. We obtained a higher contrast image with pinhole illumination (Fig. 6*c*) than without the pinhole (Fig. 6*b*). Mononucleated myoblasts were indicated with arrows, and the early phase of fused myotubes was marked with *asterisks*. We took the photograph with the Smartphone selfie camera.

We observed 1-day cultured cells with the “inverted Smartphone microscope.” We compared the effect of pinhole illumination (Fig. 5*c*). As a control, we observed the same areas of cells with an inverted phase-contrast microscope, Nikon TMD (Fig. 5*a*). As a result, the pinhole illumination improved the contrast and sharpness of the images. The cell images were clear enough to distinguish unfused myoblasts (indicated with arrows in Fig. 5*c*) and aligned myoblasts (marked with asterisks in Fig. 5*c*) at the early phase of myotube formation. Bar indicated 100 µm.

### Muscle cells observed under a standard upright microscope

**Figure 6.**
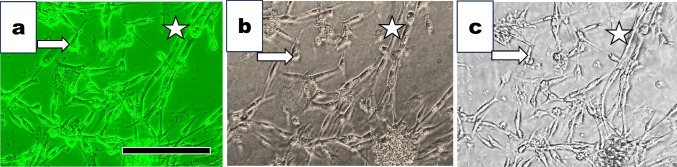
Observation of muscle cells cultured for 2 d under three microscopies. We observed cells cultured for two d under three distinct microscopies. (*a*) The cells observed under the inverted phase-contrast microscope. We saw spindle-shaped unfused single myoblasts marked with arrows and fused multinucleated myotubes marked with *asterisks*. (*b*) The flask flipped over, upside-down, and was placed on the stage. We observed cells in the same area shown in Fig. 6*a* under the conventional microscope without phase-contrast optics. (*c*) The cells in the same area in Fig. 6*b* were observed under the inverted smartphone microscope. Cells in the culture flask illuminated with an LED lamp through a pinhole were shown. Simple Camera software-installed Smartphone, Apple 11Pro, was used. The *bar* indicates 100 µm.

On 2 days in culture, unfused myoblasts (indicated with asterisks in Fig. 6*a*, *b*, *c*) and premature myotubes (marked with arrows in Fig. 6*a*, *b*) were observed under an inverted phase-contrast microscope (Fig. 6*a*) and a standard upright microscope (Fig. 6*b*). Cells in the same areas of Fig. 6*a* and *b* were observed under the inverted Smartphone microscope (Fig. 6*c*). Although the image obtained with the inverted phase-contrast microscope was slightly better, the images obtained with the latter two microscopes were good enough to see the muscle cell differentiation at a single-cell level. We could obtain cell images sufficient to study cell biology with these three distinct microscopies.

### Myotubes observed with three microscopies

We compared photographs of muscle cells cultured for 6 d under three microscopies.

**Figure 7.**
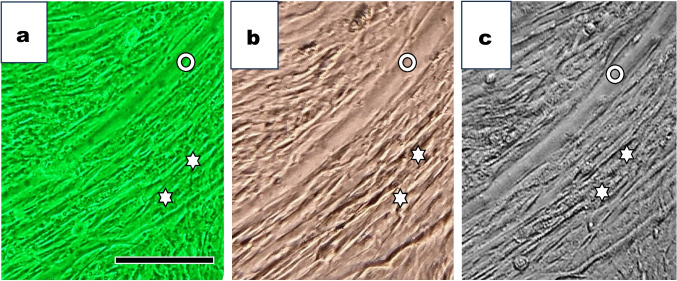
Observation of muscle cells cultured for 6 d under three distinct microscopes. (*a*) Muscle cells were cultured for 6 d and observed under an inverted phase-contrast microscope. We observed multinucleated myotubes (Indicated with a *circle*) and surrounding single cells (Indicated with an *arrow*). (*b*) The flask flipped over and upside down, and cells in the same area in (*a*) were observed under a standard upright microscope. We saw multinucleated myotubes and surrounding single cells. (*c*) Cells in the same area shown in (*b*) were observed under the inverted Smartphone microscope. Simple Camera software-installed smartphone, Apple 11Pro, was used. We took the photograph with the smartphone selfie camera. The *bar* indicates 100 µm.

Images obtained from an inverted phase-contrast microscope (Fig. 7*a*), a standard upright microscope (Fig. 7*b*), and an inverted Smartphone microscope (Fig. 7*c*) were shown. A large myotube (circles in Fig. 7*a*, *b*, c) and surrounding fibroblasts (asterisks in Fig. 7a, *b*, *c*) were visible.

**Figure 8.**
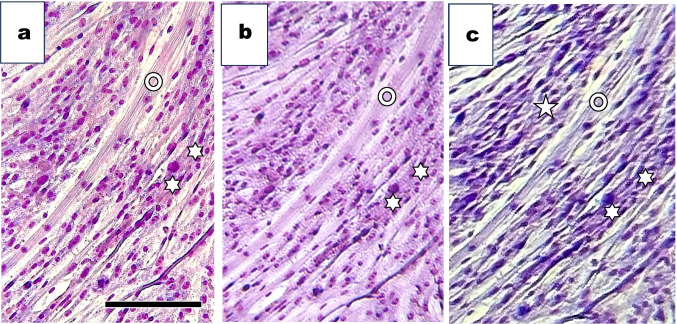
Giemsa’s Azur eosin methylene blue–stained muscle cells cultured for 6 d under three distinct microscopies. (*a*) After staining, we observed the same areas of cells shown in Fig. 7 under an inverted phase-contrast microscope. (*b*) After staining, we flipped the flask over upside-down. We observed the same area of cells in Fig. 7 under a standard upright microscope. (*c*) After staining, we observed the same area of the cells shown in Fig. 7 under the inverted smartphone microscope. We used a Simple Camera software-installed smartphone, Apple 11Pro, Bar=100 µm.

By staining cells with Giemsa’s Azur eosin methylene blue, nuclei in the myotube were visible under the three microscopies. Images obtained from an inverted phase-contrast microscope (Fig. 8*a*), a standard upright microscope (Fig. 8*b*), and an inverted Smartphone microscope (Fig. 8*c*) were shown. Circle indicated myotube, and asterisks indicated fibroblast areas.

### Observation of skeletal muscle explant prepared and photographed by high school students

**Figure 9.**
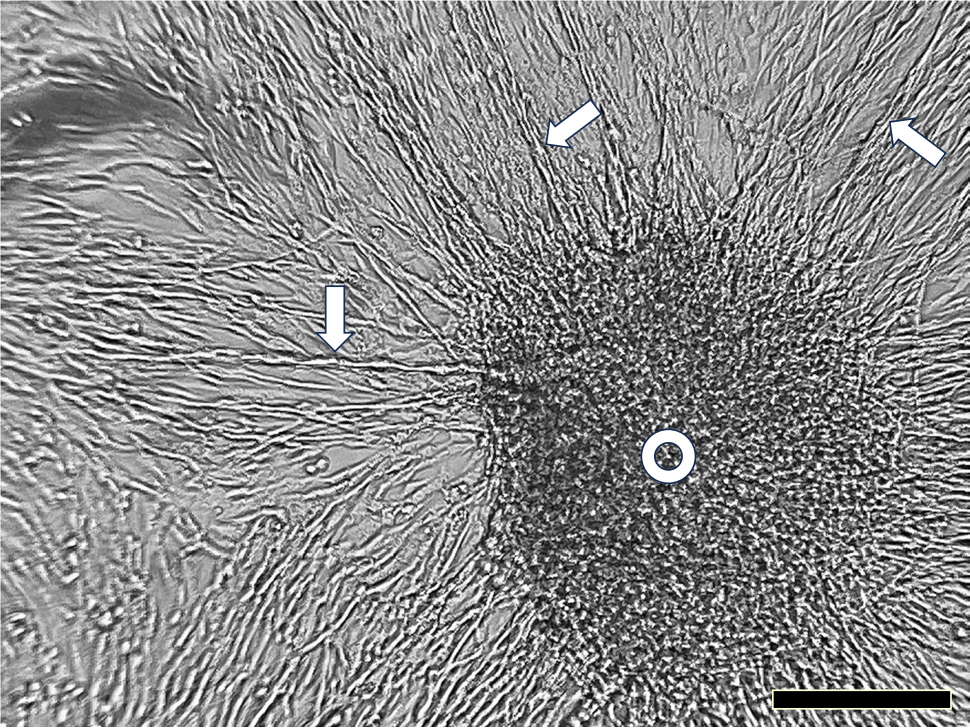
Observation of muscle explant culture, prepared and photographed under the inverted smartphone microscope by high school students. A muscle explant culture was prepared and cultured for 2 d and photographed by high school students through the inverted smartphone microscope. Cells migrated out radially from a muscle explant (the *right side* of the field, a dark area) and formed early myotubes indicated with *white arrows*. We used a Simple Camera software-installed smartphone, an Apple iPhone 13mini, to take photographs of the cells, Bar=100 µm.

Even after scissoring and pipetting muscle tissue pieces repeatedly, the remaining cell clamps or muscle explants were inevitable in muscle cell suspension. Therefore, we could occasionally observe the fate of muscle explants unexpectedly. During a public cell culture course, high school students cultured muscle cells in a flask for 2 d, and the same students took photographs under the inverted Smartphone microscope (Fig. 9). Myoblasts migrated radially from the explant (white circle in Fig. 9), and some aligned to form myotubes (white arrows in Fig. 9). This photograph indicated that high school students could culture and observe muscle cells using the methods described here. For students, obtaining natural living cell images in the DIY way was much better for studying cell biology than just looking at pictures in printed textbooks.

### Video recording of spontaneous contraction in 21-day cultured skeletal muscle cells

We could observe spontaneous contractions of myotubes cultured for 21 days on the flipped flask under a standard upright microscope. A high school science teacher took a video of these contracting muscle cells and uploaded a YouTube video at https://youtu.be/V5LqHvXn8ME.

### Technical concerns of embryonic chicken muscle cell culture

- You may need to obtain authorization for the use of chick embryos at school. We should reduce the number of fertilized eggs used for cell culture. It was enough to obtain breast muscle cells from one chicken embryo for over 20 flasks. It may help to reduce the number of fertilized eggs used for cell culture experiments.
- Instructors should explain to students how to terminate embryos and cultures safely.
- We cooled down the eggs in a refrigerator for 10 min before dissection to perform cold anesthesia. After finishing the cell culture experiment, we immersed flasks in a 50-times diluted Chlorox/sodium hypochlorite solution for at least 10 min. Then the Chlorox was drained into the sink with enough running water. We should not pour cultures directly down the drain or toilet.
- Alternatively, we found that dissociated cells could survive in a culture tube at 20 °C for over 1 w. Longer shelf lives of cells may provide teachers enough flexibility to conduct cell culture experiments in school.
- Aseptic procedures in the dissection of embryonic muscles and in preparation of the L-15 culture medium may be the technical bottlenecks in this cell culture experiment. Asking for support in providing dissociated cells and preparing the culture medium from experienced researchers or educational material suppliers may also be helpful.
- In our public cell culture course in the winter, we asked students to dissect chick embryos and dissociate cells without using the sterile box; we did not face contamination problems. However, when students performed dissection and cell dissociation in the summer without using the sterile box, we encountered significant bacterial and fungi contaminations due to higher levels of air-borne microorganisms in the summer than in the winter. Even in the winter, quick dissection and dissociation procedures were essential to avoid bacterial contamination.

### Impacts of cell observation in high school and college students

With the L-15 medium and air-tight culture flasks, living cells can grow and differentiate in a conventional incubator. We could observe the cells at the single-cell level under a standard upright microscope when we flipped the culture flask upside down. We could also observe cultured cells lens under an inverted Smartphone microscope.

With these tips, culturing cells, watching cells, and taking pictures or videos of living cells in Biology class/laboratory courses in high school and college became more accessible and affordable. The images’ quality was high and almost equivalent to classic inverted phase-contrast microscopes. Students may challenge experiments on changing culture temperatures, light wavelength, frequency of vibrations, adding fungi and bacteria, adding cooking ingredients or chemicals to the media, changing the angle of culture flasks in the incubator, etc. Students can do the DIY experiments in the classroom and at home. This way, “Study nature, not books (Louis Agassiz)” can become a reality in Cell Biology classes.

## Conclusions

In the present study, we introduced easy and affordable methods to culture embryonic chicken skeletal muscle cells by using a standard dry incubator instead of a CO_2_ incubator and observing them under a standard upright microscope instead of an inverted phase-contrast microscope. We also used the laser pickup lens from a compact disk (CD) player and attached it to the Smartphone’s selfie camera to increase magnification power and resolution. The proposed inverted smartphone microscope allowed us to observe living cells at the single-cell level. It is far more critical for students to watch living cells in a “Do-It-Yourself (DIY)” way than just simply looking at pictures of cells in textbooks.
